# Hybrid Lymphovenous Anastomosis Surgery Guided by Intraoperative Mesenteric Intranodal Lymphangiography for Refractory Nontraumatic Chylous Ascites: A Case Report

**DOI:** 10.1055/s-0043-1776304

**Published:** 2024-02-28

**Authors:** Soo Jin Woo, Saebeom Hur, Hee Seung Kim, Hak Chang, Ji-Young Kim, Soo Jin Park, Ung Sik Jin

**Affiliations:** 1 Department of Plastic and Reconstructive Surgery, Seoul National University College of Medicine, Seoul, Republic of Korea; 2 Department of Radiology, Seoul National University College of Medicine, Seoul, Republic of Korea; 3 Department of Obstetrics and Gynecology, Seoul National University College of Medicine, Seoul, Republic of Korea

**Keywords:** chylous ascites, lymphangiography, lymphovenous anastomosis

## Abstract

Refractory chylous ascites can cause significant nutritional and immunologic morbidity, but no clear treatment has been established. This article introduces a case of a 22-year-old female patient with an underlying lymphatic anomaly who presented with refractory chylous ascites after laparoscopic adnexectomy for ovarian teratoma which aggravated after thoracic duct embolization. Ascites (>3,000 mL/d) had to be drained via a percutaneous catheter to relieve abdominal distention and consequent dyspnea, leading to significant cachexia and weight loss. Two sessions of hybrid lymphovenous anastomosis (LVA) surgery with intraoperative mesenteric lymphangiography guidance were performed to decompress the lymphatics. The first LVA was done between inferior mesenteric vein and left para-aortic enlarged lymphatics in a side-to-side manner. The daily drainage of chylous ascites significantly decreased to 130 mL/day immediately following surgery but increased 6 days later. An additional LVA was performed between right ovarian vein and enlarged lymphatics in aortocaval area in side-to-side and end-to-side manner. The chylous ascites resolved subsequently without any complications, and the patient was discharged after 2 weeks. The patient regained weight without ascites recurrence after 22 months of follow-up. This case shares a successful experience of treating refractory chylous ascites with lymphatic anomaly through LVA, reversing the patient's life-threatening weight loss. LVA was applied with a multidisciplinary approach using intraoperative mesenteric lipiodol, and results showed the possibility of expanding its use to challenging problems in the intraperitoneal cavity.

## Introduction

Chylous ascites are ascites that result from leakage of triglyceride-rich lymph from the mesentery into the peritoneal cavity due to obstruction or injury in the lymphatic system. Conservative treatment includes diet modification to reduce chyle flow and allow the leak to heal, but reported success rate is 71%. 1 Peritoneovenous shunts are therapeutic and palliative options for managing severe cases in patients who cannot undergo surgical exploration. 2 However, they can be associated with complications such as occlusion of the shunt, disseminated intravascular coagulation, hypokalemia. 3 Recently, image-guided percutaneous glue embolization of abnormal lymphatic vessels has been proven safe and effective for various lymphatic leakages. 4 But it is often inadequate to completely manage severe nontraumatic cases when abnormal lymphatic structures and multiple foci of leakages are extensively involved.

This is a case of a 22-year-old female with refractory chylous ascites, which was successfully resolved with a non-traditional method. A multidisciplinary approach is introduced to perform lymphovascular anastomosis (LVA) with intraoperative mesenteric lipiodol lymphangiography and fluorescence laparoscopy.

## Case



**Video 1** An intraoperative video of intranodal lymphangiography through an inguinal lymph node with indocyanine green injection to visualize the leakage point.


**Fig. 1:**
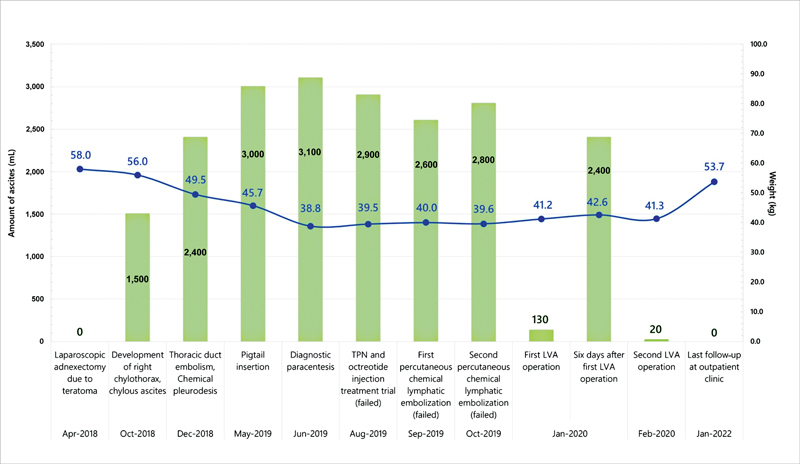
Timeline of the amount of ascites and the patient's weight since the first event. LVA, lymphovenous anastomosis.

The study protocol was approved by institutional review board. A 22-year-old female suffered from chylous ascites and chylothorax, which developed after laparoscopic left adnexectomy for a mature teratoma ( Fig. 1 ). Patient underwent thoracic duct embolization for dyspnea from right chylothorax. While chylothorax was resolved after additional chemical pleurodesis via video-assisted thoracoscopic surgery, drainage volume of chylous ascites had increased to more than 3,000 mL/day. Patient's body weight spontaneously decreased from 58 to 38 kg when referred for further treatment of refractory chylous ascites leading to significant cachexia.

Diagnostic paracentesis was performed upon admission to clarify the etiology of chylous ascites. The ascitic fluid was milky and had a triglyceride concentration of 347 mg/dL. Serum ascites albumin gradient <1.1 g/dL and was negative for malignant cytology and cultures.

**Fig. 2:**
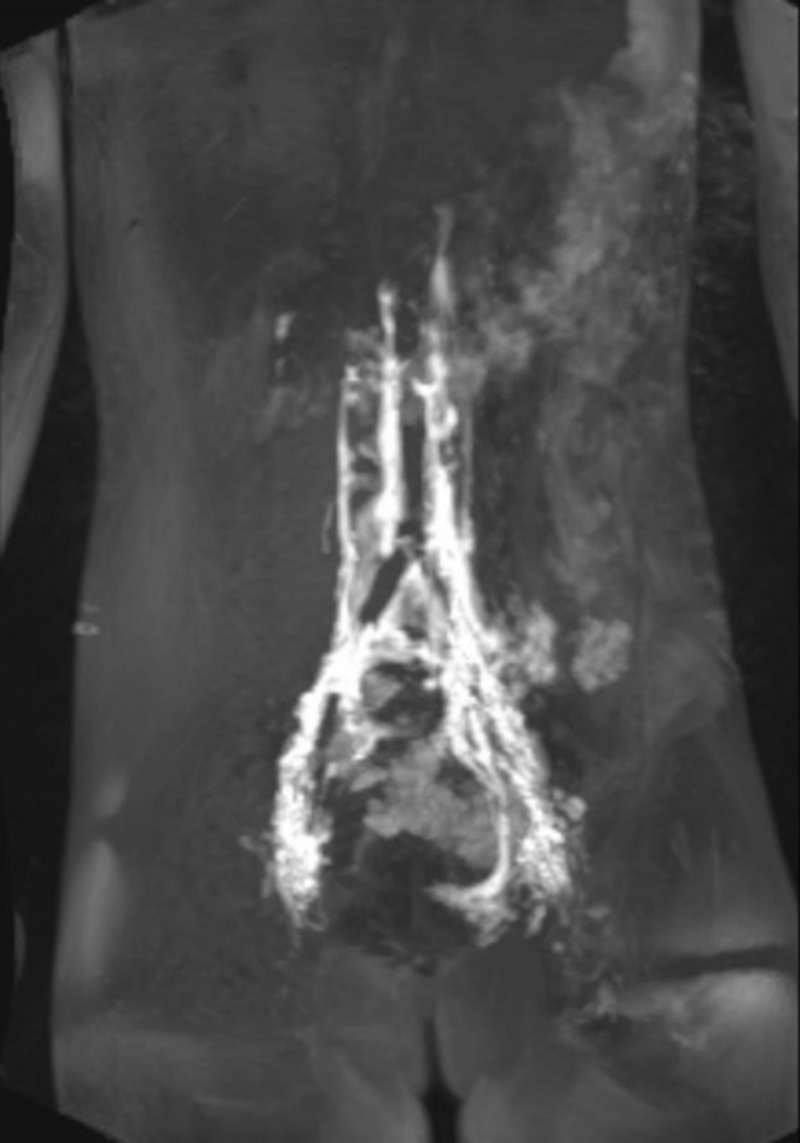
Magnetic resonance lymphangiography shows resolution of extensive enhancement ventral to the sacrum after the first embolization. Lymphatic reflux from the lymphatic vessels near the left ovarian vein is observed in the left pelvis and left salpinx. No obvious normal thoracic duct is visible.

Total parenteral nutrition and subcutaneous octreotide injection (0.1 mg/mL) were ineffective, and two times of percutaneous embolization of the dilated abnormal lymphatic vessels in pelvis failed. Magnetic resonance lymphangiography revealed prominently enhanced lymphatic vessels around left ovarian vein which were assumed to be due to abnormal reflux of the lymphatic flow resulting from obstruction of thoracic duct ( Fig. 2 ). The multidisciplinary team of interventional radiologists, gynecologic oncologists, and plastic surgery highlighted the need for a new lymphovenous shunt to relieve increased lymphatic pressure.

**Fig. 3:**
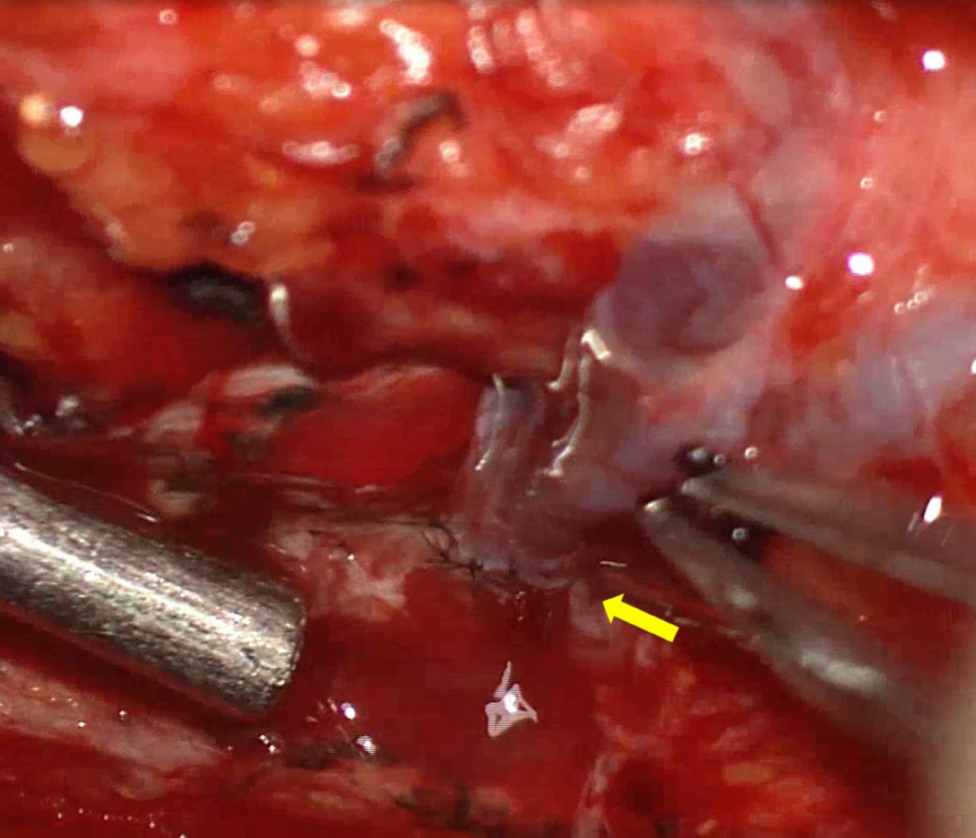
An intraoperative photograph, captured under a microscope, shows side-to-side anastomosis between lymphatics below the left para-aortic lymph node to mesenteric branch veins (arrow) during the first operation.

Gynecologic oncologists performed an exploratory laparotomy while ascites continued to fill from retroperitoneal area. Interventional radiologist punctured mesenteric lymph node under sonographic guidance and injected Lipiodol® and indigo carmine dye under C-arm to visualize the lymphatic vessel. 5 Mesenteric lymphangiography showed lymphatic fluid draining from the small bowel to not the cisterna chyli but to an enlarged lymphatic vessel running posteriorly along the left side of the lumbar vertebrae at the level of kidney. There was no definite leakage site, but multiple lymphangiectasia of the rectosigmoid colon and blue dye deposition near the left ovarian vein were visible ( Supplementary Fig. S1 , available in online version). Plastic surgeon performed LVA with 9–0 nylon in a side-to-side manner to decompress lymphatic pressure between the engorged lymphatics distal to the left para-aortic lymph node and branch of inferior mesenteric vein ( Fig. 3 ).

**Fig. 4:**
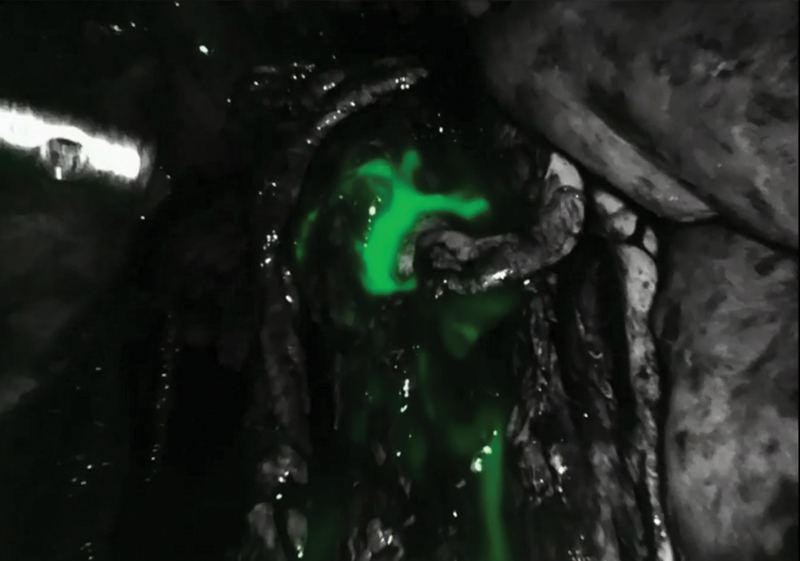
Intraoperative intranodal lymphangiography through an inguinal lymph node was performed with indocyanine green injection through the right inguinal lymph node to visualize the residual leakage point. The left iliac lymph node is intact, but leakage from the retroperitoneal lymphatics is observed.

**Fig. 5:**
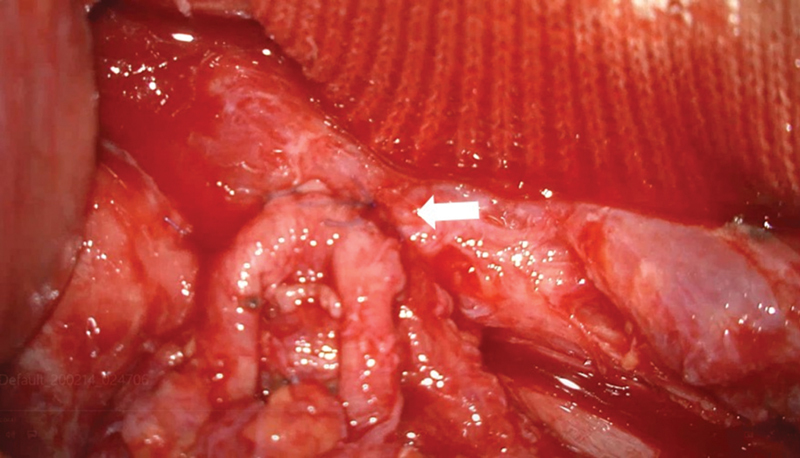
An intraoperative photograph, captured under a microscope, shows side-to-side (arrow) leakage between the right ovarian vein and the pseudo lumen of the para-aortic engorged lymphatic tissue during the second operation.

The volume of chylous ascites drainage decreased to 130 mL/day immediately although the improvement lasted only 6 days before an abrupt increase ( Supplementary Fig. S2 , available in online version). The team decided that a clearer visualization of the leakage site was required to prevent recurrence. Therefore, the patient was prepared by feeding fat fluid before surgery to create an additional lymphovenous shunt and embolize the abnormal leaking lymphatic vessels in the pelvis. Chyle leakage was observed from the lymphatics of the left para-aortic area and at multiple locations in the right aortocaval area. Interventional radiologist embolized the abnormally dilated lymphatic vessels in the pelvic wall using glue and Lipiodol mixture ( Supplementary Fig. S3 , available in online version). Intraoperative inguinal intranodal lymphangiography with indocyanine green revealed additional leakage point from the retroperitoneal lymphatics ( Fig. 4 ; Video 1 ). The plastic surgeon created additional LVA at two sites between the right ovarian vein and the pseudo lumen of the engorged para-aortic lymphatic tissue ( Fig. 5 ).

The patient was discharged 2 weeks after the second surgery and the drainage had decreased to approximately 20 mL/day. A gene study revealed c.7583C > T, p.Pro2528 Leu variant, inducing heterozygous mutations in *CELSR1* gene. The patient has had no recurrence of ascites during the last 22 months of follow-up.

## Discussion

Chylous ascites occurs when chyle-rich lymph formed by fat absorption at the intestine leaks from anywhere along its downstream pathway to thoracic duct. It results in loss of large volume of chyle, leading to hypoalbuminemia, malnutrition, and increased mortality. It is roughly categorized into traumatic and nontraumatic. Lymphatic anomalies and malignancy were most frequent causes of nontraumatic chylous ascites. 6

The exact cause and sequence of the etiology in this patient is not clear although inferences can be drawn that this patient had severe underlying lymphatic anomaly. The first clue is mutations in *CELSR1* gene which was also found in the patient's father's sample. Maltese et al reported that 5 patients out of 95 (5.3%) with primary lymphedema involving lower extremities, presented with *CELSR1* loss-of-function variants with variable phenotypic expression and incomplete penetrance. 7 Next clue is the extensive lymphatic abnormalities in the chest, shown in the preoperative imaging ( Supplementary Fig. S4 , available in online version). Initial surgery to remove the teratoma could have made an iatrogenic injury to lymphatic vessels, as the incidence of postoperative chylous ascites in gynecological surgery ranges from 0.17 to 4%. 8 9 10 Another factor to be considered is the effect of thoracic duct embolization. The blockage of lymphatic flow through thoracic duct is reported to result in complications related to redistribution of overall flow, causing leg swelling 11 and chylous ascites. 12 13

Unlike traumatic cases, interventional procedures such as lymphangiography and thoracic embolization for diagnosis and treatment of chylous ascites, are unsatisfactory. According to Nadolski's report of 31 patients (25 traumatic, 6 nontraumatic) with chylous ascites, only 55% of patients (15 trauma patients, 2 nontraumatic patients) had an outflow site confirmed on lymphangiography. 14 In remaining 45% of patients whose outflow site was not confirmed, only 21% showed decrease in leakage after treatment with Lipiodol or spontaneous recovery. This is because, in current lymphatic imaging, contrast agent is injected into central lymphatic system through the lymphatic system of the lower extremities. It is near cisterna chyli that chyle is joined by hepatic and intestinal lymphatic systems, and then returns to the veins via the thoracic duct. Therefore, in cases of chylous ascites, most of the lymphatic system of the liver and intestinal tract is upstream of flow, so contrast cannot be seen without diffusion or reflux. In such cases, contrast medium cannot reach the leakage site in chylous ascites by inguinal lymphangiography but can only be identified through intraoperative mesenteric lymphangiography. The requirement for open laparotomy to allow sonographic guidance during mesenteric lymph node puncture limits its use to cases without other treatment options.

Although there is no standard treatment protocol for refractory chylous ascites, microsurgical advancements have provided new surgical options. Arakaki et al reported performing LVA around ankle, which successfully resolved leakage for the left lumbar lymph trunk in a patient who underwent total abdominal hysterectomy, bilateral salpingo-oophorectomy, and pelvic lymph node dissection for tubal cancer. 15 Weissler reported effective management with LVA in managing recalcitrant chylothoraces secondary to thoracic duct injury in two infants who underwent surgical intervention for congenital cardiac anomalies; LVA restored normal lymphatic circulation within 1 to 2 weeks. 16

Due to more aggressive retroperitoneal surgery and longer survival of patients with cancer, incidence of chylous ascites is increasing. 17 Although the manuscript illustrates a single case with a short-term follow-up period, it presents the possibility of expanding the scope of LVA surgery in managing chylous ascites in patients with congenital lymphatic anomaly resistant to conservative, radiological, or surgical procedures. LVA was introduced to the intraperitoneal space and proved an effective option to liberate the patient from repeated paracentesis and reverse the life-threatening weight loss. Further research and evaluation on efficacy of LVA for refractory chylous ascites are required with more cases and longer follow-up periods to provide clinical guidelines.

## References

[1] Weniger M D'Haese J G Angele M K Kleespies A Werner J Hartwig W Treatment options for chylous ascites after major abdominal surgery: a systematic review Am J Surg 2016 211 01 206 213 26117431 10.1016/j.amjsurg.2015.04.012

[2] Leibovitch I Mor Y Golomb J Ramon J The diagnosis and management of postoperative chylous ascites J Urol 2002 167(2 Pt 1):449 457 11792897 10.1016/S0022-5347(01)69064-5

[3] Manolitsas T P Abdessalam S Fowler J M Chylous ascites following treatment for gynecologic malignancies Gynecol Oncol 2002 86 03 370 374 12217764 10.1006/gyno.2002.6754

[4] Hur S Jun H Jeong Y S Novel interventional radiological management for lymphatic leakages after gynecologic surgery: lymphangiography and embolization Gland Surg 2021 10 03 1260 1267 33842273 10.21037/gs-2019-ursoc-10 PMC8033090

[5] Lee H Kim S J Hur S The feasibility of mesenteric intranodal lymphangiography: its clinical application for refractory postoperative chylous ascites J Vasc Interv Radiol 2018 29 09 1290 1292 30146199 10.1016/j.jvir.2018.01.789

[6] Steinemann D C Dindo D Clavien P-A Nocito A Atraumatic chylous ascites: systematic review on symptoms and causes J Am Coll Surg 2011 212 05 899 905.e1,4 21398159 10.1016/j.jamcollsurg.2011.01.010

[7] Maltese P E Michelini S Ricci M Increasing evidence of hereditary lymphedema caused by CELSR1 loss-of-function variants Am J Med Genet A 2019 179 09 1718 1724 31215153 10.1002/ajmg.a.61269

[8] Frey M K Ward N M Caputo T A Taylor J Worley M J Jr. Slomovitz B M Lymphatic ascites following pelvic and paraaortic lymphadenectomy procedures for gynecologic malignancies Gynecol Oncol 2012 125 01 48 53 22101154 10.1016/j.ygyno.2011.11.012

[9] Tulunay G Ureyen I Turan T Chylous ascites: analysis of 24 patients Gynecol Oncol 2012 127 01 191 197 22728517 10.1016/j.ygyno.2012.06.023

[10] Zhao Y Hu W Hou X Zhou Q Chylous ascites after laparoscopic lymph node dissection in gynecologic malignancies J Minim Invasive Gynecol 2014 21 01 90 96 23900043 10.1016/j.jmig.2013.07.005

[11] Laslett D Trerotola S O Itkin M Delayed complications following technically successful thoracic duct embolization J Vasc Interv Radiol 2012 23 01 76 79 22115569 10.1016/j.jvir.2011.10.008

[12] Le Pimpec-Barthes F Pham M Jouan J Bel A Fabiani J-N Riquet M Peritoneoatrial shunting for intractable chylous ascites complicating thoracic duct ligation Ann Thorac Surg 2009 87 05 1601 1603 19379920 10.1016/j.athoracsur.2008.09.029

[13] Gaba R C Owens C A Bui J T Carrillo T C Knuttinen M G Chylous ascites: a rare complication of thoracic duct embolization for chylothorax Cardiovasc Intervent Radiol 2011 34 02 S245 S249 20517611 10.1007/s00270-010-9900-4

[14] Nadolski G J Chauhan N R Itkin M Lymphangiography and lymphatic embolization for the treatment of refractory chylous ascites Cardiovasc Intervent Radiol 2018 41 03 415 423 29238869 10.1007/s00270-017-1856-1

[15] Arakaki Y Shimoji Y Yamazaki S Shimizu Y Aoki Y Microsurgical lymphaticovenular anastomosis for refractory chylous ascites following para-aortic lymph nodes dissection in a patient with tubal cancer Gynecol Oncol Rep 2018 26 53 55 30302363 10.1016/j.gore.2018.09.004 PMC6174838

[16] Weissler J M Cho E H Koltz P F Lymphovenous anastomosis for the treatment of chylothorax in infants: a novel microsurgical approach to a devastating problem Plast Reconstr Surg 2018 141 06 1502 1507 29794709 10.1097/PRS.0000000000004424

[17] Aalami O O Allen D B Organ C H Jr Chylous ascites: a collective review Surgery 2000 128 05 761 778 11056439 10.1067/msy.2000.109502

