# Supplementary material for: Hybrid Lymphovenous Anastomosis Surgery Guided by Intraoperative Mesenteric Intranodal Lymphangiography for Refractory Nontraumatic Chylous Ascites: A Case Report
Source: Arch Plast Surg. 2024 Feb 28;51(1):130–4. doi: 10.1055/s-0043-1776304 (PMC10901586; doi:10.1055/s-0043-1776304)
Supplement: Supplementary file 2 — Supplementary Material [file 10-1055-s-0043-1776304-s23jul0413cr.pdf]

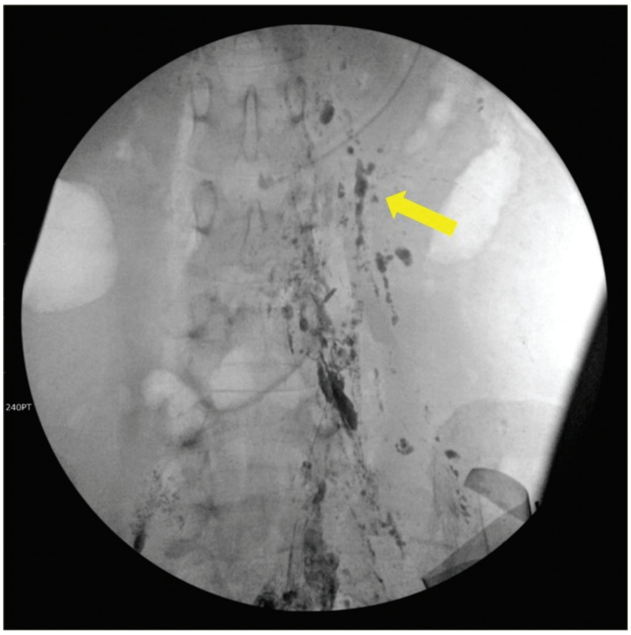

Supplementary Fig. 1 Lymph node parenchyma and efferent vessels were contrasted using a water-soluble contrast agent by puncturing mesenteric lymph node under ultrasound guidance. Then Lipiodol 5cc and indigo carmine (50% dilution in normal saline, 10 ml) were injected to perform intraoperative mesenteric lymphangiography. It shows enlarged lymphatic vessel running downwards from the left side of the lumbar vertebra around the ovarian vein (arrow).

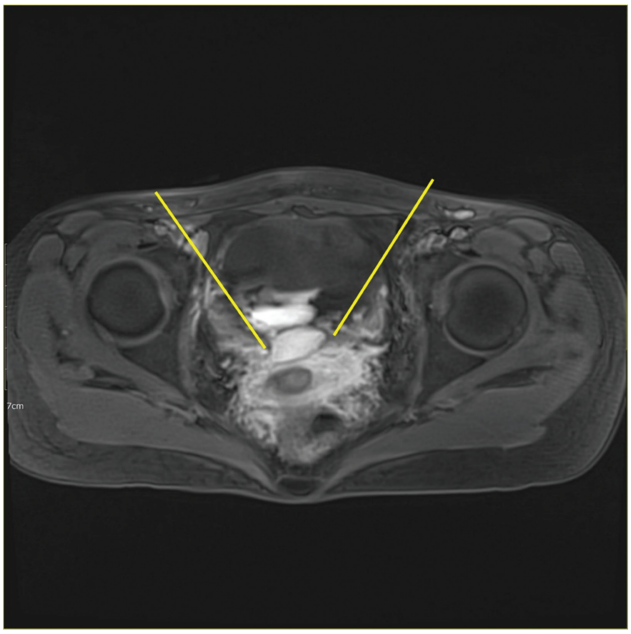

Supplementary Fig. 2 Magnetic resonance lymphangiography after the first operation showing redistribution of retroperitoneal lymphatic flow with more dilated retroperitoneal lymphatic structures in the pelvic retroperitoneum and extravasation of contrast agent mainly around the uterus.

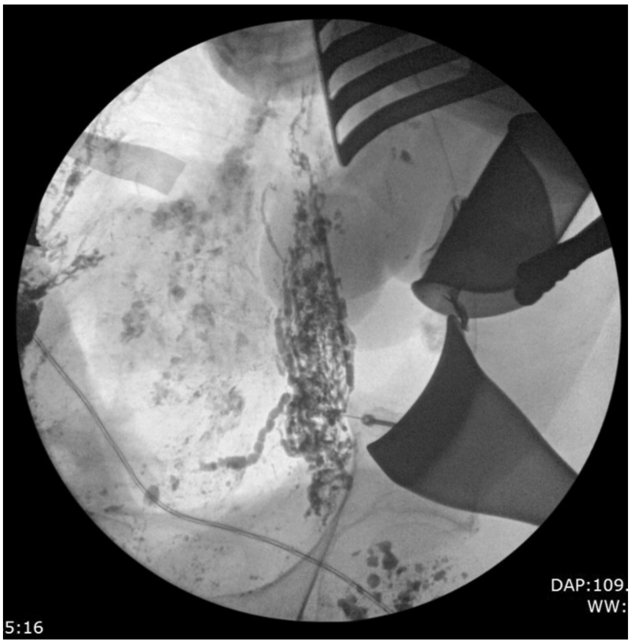

Supplementary Fig. 3 During the second operation, lymphatics were punctured with a 26G needle through both iliac veins under ultrasound guidance and embolization was performed with 10 ml of 1:3 glue:Lipiodol.

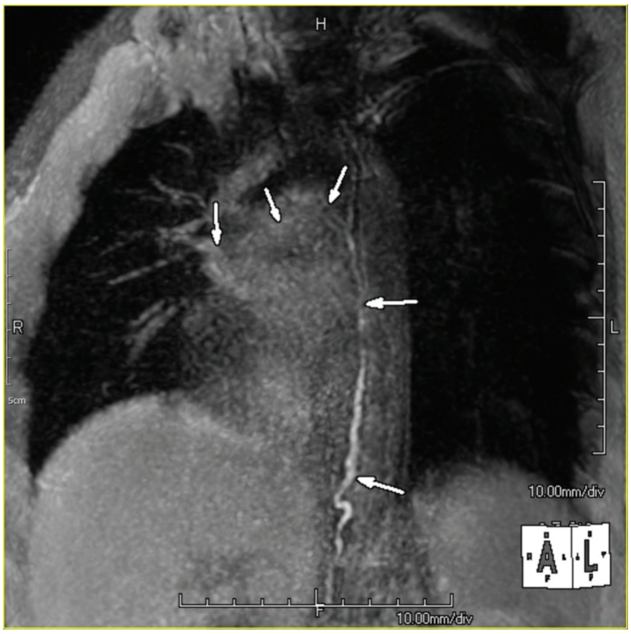

Supplementary Fig. 4 An enlarged diameters and tortuous configuration of the thoracic duct of this patient shown in T2 weighted MR image.

 © 2024. The Author(s).
